# Use of psychotropic drugs before pregnancy and the risk for induced abortion: population-based register-data from Finland 1996-2006

**DOI:** 10.1186/1471-2458-10-383

**Published:** 2010-06-30

**Authors:** Mika Gissler, Miia Artama, Annukka Ritvanen, Kristian Wahlbeck

**Affiliations:** 1 THL National Institute for Health and Welfare, Helsinki, Finland; 2 Nordic School of Public Health, Gothenburg, Sweden; 3 Vaasa Central Hospital, Department of Psychiatry, Vaasa, Finland

## Abstract

**Background:**

Some, though not all studies have reported an increased risk for mental health problems after an induced abortion. Problems with design and data have compromised these studies and the generalisation of their results.

**Methods:**

The Finnish Medication and Pregnancy database (N = 622 671 births and 114 518 induced abortions for other than fetal reasons) in 1996-2006 was utilised to study the use of psychotropic drugs in the three months before a pregnancy ending in a birth or an induced abortion.

**Results:**

In total 2.1% of women with a birth and 5.1% of women with an induced abortion had used a psychotropic medicine 0-3 months before pregnancy. Psychotropic drug users terminated their pregnancies (30.9%) more often than other pregnant women (15.5%). Adjustment for background characteristics explained one third of this elevated risk, but the risk remained significantly increased among users of psychotropic medicine (OR 1.94, 95% confidence intervals 1.87-2.02). A similar risk was found for first pregnancies (30.1% vs. 18.9%; adjusted OR 1.53, 95% confidence intervals 1.42-1.65). The rate for terminating pregnancy was the highest for women using hypnotics and sedatives (35.6% for all pregnancies and 29.1% for first pregnancies), followed by antipsychotics (33.9% and 36.0%) and antidepressants (32.0% and 32.1%).

**Conclusions:**

The observed increased risk for induced abortion among women with psychotropic medication highlighs the importance to acknowledge the mental health needs of women seeking an induced abortion. Further studies are needed to establish the impact of pre-existing differences in mental health on mental health outcomes of induced abortions compared to outcomes of pregnancies ending in a birth.

## Background

The connection of induced abortion and subsequent mental health problems is controversial. Studies from countries with free or relatively liberal abortion legislation have suggested that women experiencing an induced abortion have a higher risk for depression [1-5], anxiety [2,5-10], suicide [11,12], suicidal behaviour [2], substance use disorders [2], and any mental health problem requiring outpatient treatment [13] after the procedure. In most of these studies, the available confounding factors did not explain the elevated risk for harmful outcomes.

Some other studies, however, have reported no elevated risk for any psychiatric disorder [14], anxiety [7,15] or depression [1,5,16,17] among women who had experienced induced abortion. In Israel, pregnancy termination has been shown to result in a significant reduction in the pre-abortion psychological distress level [18]. A Swedish study reported no suffering or any subsequent regret or negative effects one year after termination [19]. Studying welfare more broadly, a study from New Zealand reported that young women who became pregnant before age 21 and terminated their pregnancy had even better outcomes on education, income, welfare dependence and domestic violence compared to young women who did not seek an abortion [20]. A recent review [21] concluded that in 21 eligible studies on the connection of induced abortion and long-term mental health outcomes, 8 reported neutral, 6 negative, and 7 mixed findings.

Two reviews have concluded that studies on post-abortion mental health problems often face significant problems related to their design or data [21,22]. Terminated pregnancies are usually compared with pregnancies ending in a birth, but it would be essential to disaggrate them by pregnancy intention and to analyse intended and unintended (i.e. unwanted and mistimed) pregnancies separately. Information on pregnancy intention prior to pregnancy is, however, seldom available. Retrospective collection of such data may be biased, and the data remains unavailable in routinely collected databases.

Another major problem is that the mental health measurement is not always based on validated diagnostic instruments [21], which can be collected either prospectively or retrospectively from medical records and by using surveys. In register studies, the mental health measurements have been based on physician's diagnosis on inpatient or outpatient care [13] or, in case of suicide, on main cause of death [11,12].

The third major problem is how confounders are controlled. In several studies, the number of background variables describing common determinants of mental health has been limited, and important determinants have remained unavailable. Information on pre-existing mental health status has seldom been available, even though it has been shown to be an important explanatory factor for the increased risk for negative mental health outcomes in several [7,14], though not in all studies [16]. Since induced abortion and mental health problems share same risk factors - such as young age [12], poor socioeconomic circumstances, violence [3,7], and rape history [7] - the causality between induced abortion and subsequent mental health problems cannot be straightforwardly concluded without taking into consideration their common determinants.

Our aim was to clarify the pre-existing mental health status among pregnant women by using the Medication and Pregnancy database with information on all births and induced abortions in Finland in 1996-2006. We used the purchase of psychotropic drugs in the three months before pregnancy as a proxy measure of mental health status and calculated the risk for terminated pregnancy among women purchasing such drugs compared to other women with no purchases. First pregnancies were separately analysed to avoid bias caused by previous pregnancies. Selective terminations of pregnancy due to fetal anomalies were excluded, since these are originally wanted pregnancies, unlike pregnancies ending in an induced abortion in general.

## Methods

The nationwide Finnish Medication and Pregnancy database was utilised in this study. The data on births were based on the Medical Birth Register (MBR) and data on induced abortion on the Register on Induced Abortions and Sterilisations. Both of these registers are maintained by the National Institute for Health and Welfare (THL).

1) MBR. All live births and stillbirths of fetuses with a gestational age of 22 weeks or more or with a birth weight of 500 grams or more have been included in the register since 1987. The register data are collected from all delivery hospitals and sent to THL. The register includes the mother's and the child's personal identification numbers, maternal obstetrical and social background, health care and interventions during pregnancy and delivery, as well as the newborn's outcome until the age of seven days. Data quality studies have shown that the majority of the register content corresponded well or satisfactorily with hospital record data [23].

2) Register on Induced Abortions. This register collects data on all induced abortions performed in Finland since 1977 [24]. National legislation requires permission and an indication for terminating a pregnancy. An induced abortion can be perfomed for social reasons (age less than 17 years or 40 years or more at conception, delivered four children or more, or social circumstances), medical reasons (health of the pregnant woman, suspected or confirmed congenital anomaly or birth defect, or mother's and/or father's incapability to take care of the child) or ethical reasons (rape or incest). The legislation on induced abortions is interpreted liberally, and 97% of the pregnancy terminations during the study period were performed on social reasons. If the permission for an induced abortion is not granted, a national board will assess the case and grant the permission unless the gestational age limit of 20 weeks (24 weeks in case of confirmed congenital anomaly or birth defect) has not been passed.

Once the pregnancy has been terminated, a notification is sent to THL within one month of the procedure. The register includes women's personal identification number, information on women's background, the procedure, and complications occurring at the hospital. According to a 1993 data quality study, more than 99% of induced abortions mentioned in hospital records were reported to the register, and for most variables, agreement between the register data and the hospital record was good [24].

By using woman's personal identification number, pregnancy data was linked to information on all reimbursed psychotropic drugs, defined as Anatomic Therapeutic Classification (ATC) categories N05 and N06 purchased in the three months prior to estimated start of pregnancy.

3) The information on drugs was retrieved from the Register on Reimbursement Drugs, established in 1994 by the Social Insurance Institution. It includes information on the purchases of drugs that have been reimbursed under the health insurance scheme, which covers virtually all drug prescriptions for all residents of Finland. The register does not include information on OTC medicines, nor medicines used in hospitals and a few non-reimbursable drugs. The information is collected from all pharmacies, and the register includes information on purchaser, drug, dispensing and prescribing physician. Information on the purchased drug includes the Nordic Commodity Number (trade name, pharmaceutical form, strength, pack size, number of DDDs per package, marketing authorisation holder, and ATC code), number of packs received, price, reimbursement, and date of prescribing [25].

The Finnish Medication and Pregnancy database for 1996-2006 included information on 622 671 pregnancies ending in a birth and 117 229 induced abortions. We separately analysed 243 788 first pregnancies (197 120 births and 46 668 induced abortions) to avoid any bias caused by previous pregnancies and their end-point (birth, induced abortion or spontaneous abortion). Induced abortions due to fetal reasons were excluded (N = 2711 and 704, respectively).

We calculated the number of terminated pregnancies per one hundred ended pregnancies (ending in a birth or an induced abortion) for total pregnancies and by the following maternal characteristics:

- year of birth or induced abortion (from 1996 to 2006)

- age at birth or induced abortion (less than 20, 20-24, 25-29, 30-34, 35-39, 40 years or more)

- region (21 central hospital districts)

- marital status (married, cohabiting, single, unknown)

- previous pregnancies (0, 1, 2, 3 or more, unknown)

- previous births (0, 1, 2, 3 or more, unknown)

- previous induced abortions (0, 1 or more, unknown) and

- socioeconomic position based on woman's occupation (upper-white collar worker, lower-white collar worker, blue-collar worker, student, unknown).

The following ATC categories were analysed:

- N05 or N06: any tranquilising, antidepressant or psychostimulant drug

- N05A: antipsychotics

- N05B: anxiolytics

- N05C: hypnotics and sedatives

- N06A: antidepressants

- N06B: psychostimulants and nootropics and

- N06C: psycholeptics and psychoanaleptics in combination.

Results for the ATC group N06B are not shown in the Tables due to the low rate of prescription and the small number of pregnancies (N = 28). The odds ratios (OR) for a pregnancy ending in an induced abortion were calculated with 95% confidence intervals (CI). The ORs were futher adjusted using logistic regressions by the following background variables: year, age, region, marital status and socioeconomic position.

Ethical approval: the National Institute for Health and Welfare (THL), and the Social Insurance Institution (KELA) have given their permission to use their health data for the Pregnancy and Medicine -project. The board of the Pregnancy and Medicine project approved this study. Since the final database is anonymous, no informed consent of the registered person or a statement from the research ethical board was required.

## Results

In total, 15.5 out of 100 pregnancies were terminated (Table 1). More than half of women aged less than 20 years and a quarter of women aged 40 years or more terminated their pregnancies. Single women terminated their pregnancies more than seven times more often than those who were married and more than four times more often than cohabiting, unmarried women. The connection between previous obstetrical history and termination was U-shaped, and the risk for termination was the lowest for second pregnancies and births. If the women had at least one previous induced abortion, the risk for termination more than doubled (31% vs. 13%). Students terminated one out of three pregnancies (34%), while the risk was significantly lower for blue-collar workers (14%), lower-white collar workers (9%) and upper-white collar workers (5%).

**Table 1:** Background characteristics of pregnancies ending in births and induced abortions in Finland 1996-2006

	All pregnancies			First pregnancies		
	Births	Induced abortions	Induced abortions, %	Births	Induced abortions	Induced abortions, %
N	622 671	114 518	15.5	197 120	45 964	18.9
						
Age						
Less than 20	17 517	24 798	58.5	13 202	21 016	61.3
20-24	103 343	27 997	21.3	51 647	14 801	22.2
25-29	199 249	21 532	9.7	73 937	6 073	7.6
30-34	188 793	18 989	9.1	42 969	2 574	5.6
35-39	92 810	14 646	13.5	13 235	1 120	7.7
40 or more	20 959	6 556	23.5	2 130	380	14.8
						
Marital status						
Married	376 705	23 931	5.9	95 184	1 373	1.4
Cohabiting	150 070	18 779	11.1	62 148	5 530	8.1
Single	85 033	71 805	45.7	35 675	39 061	52.2
						
Previous births						
0	257 119	58 791	18.6	-	-	-
1	206 033	20 385	9.0	-	-	-
2	97 137	21 717	18.2	-	-	-
3 or more	61 023	13 625	18.2	-	-	-
						
Previous induced abortions						
0	542 605	79 404	12.7	-	-	-
1 or more	78 214	35 092	30.8	-	-	-
						
Socioeconomic position						
Upper-white collar w.	108 957	6 270	5.4	37 597	1 632	4.1
Lower-white collar w.	236 149	23 341	9.0	69 481	5 446	7.3
Blue-collar worker	95 751	15 464	13.9	27 529	4 026	12.7
Student	55 481	28 442	33.8	28 769	20 908	42.0
Other	126 333	41 001	24.4	33 744	13 952	29.1

Excluding 2711 and 704 induced abortions due to fetal reasons.

The likelihood to terminate a first pregnancy was higher, 18.9 per 100 first pregnancies (Table 1). The maternal background risk factors were similar for all pregnancies. Compared with their subsequent pregnancies, young women aged less than 20 years (the relative risk being 25% higher), single women (+15%) and students (+54%) had a higher risk to terminate their first pregnancies. Similarly, the risk to terminate their first pregnancy was significantly decreased for women aged 30 years or more (the relative risk was 11% lower), for married or cohabiting women (-15%) and for upper and lower white-collar workers (-9%) compared to their subsequent pregnancies.

In total 2.1% of women with a birth and 5.1% of women with an induced abortion had used a psychotropic medicine 0-3 months before pregnancy. The percentages doubled in both groups, from 1.5% to 2.9% among women with a birth and from 3.9% to 7.4% among all women with induced abortion during the study period of 11 years.

By background characteristics, the use of psychotropic medicine before a pregnancy ending with a birth was increased for women aged less than 20 years and aged 35 years or more, single women, women having three or more births, women having at least one induced abortion and for blue-collar workers (Table 2). For women terminating their birth, the use of psychotropic medicine increased by age and it was also increased for single women, multiparous women, and women with repeated induced abortion. Differences by socioeconomic position were relatively small.

**Table 2:** Use of psychotropic medication 0-3 months before pregnancy per 100 women

ATC code	N05/N06		N05A		N05B		N05C		N06A		N06C	
	Births	Induced abortions	Births	Induced abortions	Births	Induced abortions	Births	Induced abortions	Births	Induced abortions	Births	Induced abortions
Users	13 327	5 951	1 233	633	3 381	1 601	1 413	780	9 940	4 667	379	110
												
Per 100 women	2.1	5.1	0.2	0.5	0.5	1.4	0.2	0.7	1.6	4.0	0.1	0.1
												
Age												
Less than 20	2.3	2.5	0.3	0.2	0.4	0.3	0.1	0.1	1.9	2.2	0.1	2.2
20-24	2.0	4.5	0.2	0.4	0.5	0.2	0.1	0.4	1.6	1.9	0.0	1.9
25-29	1.8	5.7	0.2	0.6	0.4	0.3	0.2	0.6	1.4	2.5	0.0	2.5
30-34	2.0	6.5	0.2	0.7	0.6	0.3	0.2	1.1	1.5	2.8	0.1	2.8
35-39	2.7	7.0	0.3	0.8	0.8	0.4	0.4	1.2	1.9	3.6	0.1	3.6
40 or more	3.9	6.4	0.5	0.6	1.0	0.9	0.8	1.4	2.6	7.8	0.2	7.8
												
Marital status												
Married	1.8	4.4	0.2	0.2	0.5	1.3	0.2	0.4	1.3	2.1	0.1	2.1
Cohabiting	2.3	4.5	0.2	0.2	0.5	1.2	0.2	0.5	1.8	2.8	0.0	2.8
Single	3.3	5.5	0.3	0.1	1.0	1.4	0.4	0.1	2.5	0.8	0.1	0.8
												
Previous pregnancies												
0	2.1	3.7	0.2	0.1	0.5	2.2	0.2	0.2	1.6	1.2	0.1	1.2
1 or more	2.2	6.0	0.2	0.1	0.6	1.5	0.2	0.1	1.6	0.8	0.1	0.8
												
Previous births												
0	2.2	4.4	0.2	0.1	0.5	1.7	0.2	0.2	1.7	0.9	0.1	0.9
1	1.8	6.2	0.2	0.2	0.5	4.9	0.2	0.5	1.3	2.5	0.1	2.5
2	2.3	5.7	0.2	0.2	0.6	4.7	0.2	0.4	1.7	2.5	0.1	2.5
3 or more	2.7	5.3	0.3	0.3	0.7	7.5	0.3	0.7	2.0	3.9	0.1	3.9
												
Previous induced abortions												
0	1.9	4.2	0.2	0.4	0.5	1.3	0.2	0.1	1.5	0.7	0.1	0.7
1 or more	3.5	7.1	0.4	1.0	1.1	2.9	0.5	0.3	2.6	1.5	0.1	1.5
												
Socioeconomic position												
Upper-white collar w.	1.7	4.8	0.1	5.1	0.3	15.5	0.3	1.5	1.2	8.1	0.1	8.1
Lower-white collar w.	1.9	5.1	0.2	1.4	0.5	4.3	0.2	0.4	1.4	2.3	0.1	2.3
Blue-collar worker	2.3	4.8	0.2	2.2	0.7	6.6	0.2	0.6	1.6	3.5	0.1	3.5
Other	2.6	5.2	0.3	0.5	0.6	1.5	0.2	0.1	2.0	0.8	0.1	0.8

Excluding 2711 induced abortions due to fetal reasons.

N05/N06 Any in ATC categories N05 and N06

N05A Antipsychotics

N05B Anxiolytics

N05C Hypnotics and sedatives

N06A Antidepressants

N06B Psychostimulants and nootropics

N06C Psycholeptics and psychoanaleptics in combination

Among women having used psychotropic medication before pregnancy, 30.9 out of 100 pregnancies were terminated, giving a 2.5-fold risk compared to women not using such medication (Table 3). Pregnancy termination and use of psychotropic medicine shared common risk factors, and the adjustment for background variables explained slightly more than one third of the excess risk. Even after the background standardisation, however, the risk for women having used a psychotropic medicine before pregnancy to end in a pregnancy termination was significantly increased (adjusted OR = 1.94, 95% confidence interval 1.87-2.02).

**Table 3:** The proportion of terminated pregnancies among women using psychotropic medicine 0-3 months before pregnancy, Finland 1996-2006

ATC code	N05/N06	N05A	N05B	N05C	N06A	N06C
Terminated pregnancies per 100	30.9	33.9	32.1	35.6	32.0	22.5
						
Age						
Less than 20	60.3	53.6	47.0	64.2	62.0	35.3
20-24	37.6	41.7	11.8	46.0	24.2	19.6
25-29	26.0	31.8	6.8	29.8	16.2	13.5
30-34	25.0	30.0	5.7	34.1	16.4	17.5
35-39	29.5	31.7	8.1	33.0	23.6	30.9
40 or more	35.5	28.9	22.3	38.0	50.3	32.1
						
Marital status						
Married	13.9	6.2	15.9	12.0	9.8	11.7
Cohabiting	20.5	12.3	22.7	22.1	16.9	18.4
Single	58.5	12.4	56.2	22.7	20.7	55.0
						
Previous pregnancies						
0	30.1	9.5	53.6	20.2	14.9	17.9
1 or more	31.2	4.7	29.6	8.8	7.4	24.3
						
Previous births						
0	31.4	6.6	43.2	13.7	11.1	0.0
1	26.4	11.3	52.7	21.1	16.5	0.0
2	36.2	19.7	62.7	29.8	25.1	28.7
3 or more	31.2	20.4	69.8	33.9	31.2	31.4
						
Previous induced abortions						
0	24.6	26.7	29.4	8.7	6.4	12.2
1 or more	47.7	55.0	54.6	20.4	21.3	0.0
						
Socioeconomic position						
Upper-white collar worker	15.1	76.1	74.3	25.3	29.4	8.4
Lower-white collar worker	21.4	47.2	46.3	17.8	14.0	25.3
Blue-collar worker	25.6	62.1	61.0	29.3	25.9	23.9
Other	43.2	39.4	47.5	18.4	12.7	26.3
						
Crude OR	2.51	2.80	2.60	3.02	2.62	1.58
95% CI	2.43-2.59	2.55-3.08	2.45-2.76	2.76-3.29	2.53-2.71	1.28-1.96
						
Adjusted OR1)	1.94	1.85	2.02	2.25	2.00	1.69
95% CI	1.87-2.02	1.65-2.08	1.88-2.16	2.02-2.49	1.92-2.09	1.32-2.17
						
Explanation, %	37	53	36	38	38	< 0

Excluding 2711 induced abortions due to fetal reasons.

1) Adjusted by year, age, region, marital status, socioeconomic position and gravidity.

By type of medication, the highest risk for terminating pregnancy was observed for women using hypnotics and sedatives (35.6%, adjusted OR = 2.25, 95% CI 2.02-2.49), antipsychotics (33.9%, adjusted OR = 1.85, 95% CI 1.65-2.08) and antidepressants (32.0%, adjusted OR = 2.00, 95% CI 1.92-2.09).

For first pregnancies, the share of terminated pregnancies (30.1 per 100) among women using psychotropics and their risk (adjusted OR 1.53, 95% CI 1.42-1.65) were slightly smaller than for all pregnancies (Table 4). By type of medication, the highest risk for terminating pregnancy was found for women using antipsychotics (36.0%, adjusted OR = 1.73, 95% CI 1.39-2.16), antidepressants (32.1%, adjusted OR = 1.57, 95% CI 1.44-1.71), and hypnotics and sedatives (29.1%, adjusted OR = 1.74, 95% CI 1.36-2.22).

**Table 4:** The proportion of terminated pregnancies among primigravidas using psychotropic medicine 0-3 months before pregnancy, Finland 1996-2006

ATC code	N05/N06	N05A	N05B	N05C	N06A	N06C
Terminated pregnancies per 100	30.1	36.0	25.7	29.1	32.1	17.9
						
Age						
Less than 20	62.4	56.0	47.5	64.9	64.0	30.0
20-24	36.5	44.8	32.1	43.5	38.1	26.9
25-29	20.8	29.5	22.6	24.6	21.3	9.3
30-34	15.9	25.9	15.7	14.5	16.9	6.3
35-39	19.8	28.6	22.0	22.1	20.6	27.8
40 or more	22.5	4.3	20.0	27.9	23.6	60.0
						
Marital status						
Married	3.6	6.5	3.9	4.1	3.6	0.0
Cohabiting	14.8	21.3	13.6	14.8	14.8	11.8
Single	58.0	59.1	51.4	55.2	60.4	52.6
						
Socioeconomic position						
Upper-white collar worker	9.3	10.8	5.4	5.6	10.9	3.1
Lower-white collar worker	16.6	21.5	14.6	22.7	16.6	18.0
Blue-collar worker	19.7	25.0	17.9	18.0	21.8	6.7
Other	44.5	46.3	41.0	45.3	45.9	35.1
						
Crude OR	1.88	2.42	1.49	1.76	2.07	0.94
95% CI	1.78-2.00	2.05-2.86	1.31-1.69	1.47-2.12	1.94-2.20	0.60-1.46
						
Adjusted OR^1)^	1.53	1.73	1.46	1.74	1.57	1.40
95% CI	1.42-1.65	1.39-2.16	1.25-1.75	1.36-2.22	1.44-1.71	0.79-2.53
						
Explanation, %	40	49	1	4	47	< 0

Excluding 704 induced abortions due to fetal reasons.

1) Adjusted by year, age, region, marital status and socioeconomic position.

## Discussion

Our data included information on more than 737 000 pregnancies and several background variables. The data were derived from compulsory nationwide, population-based registers with high completeness and quality [23,24]. It did not, however, cover all pregnancies, since register information on pregnancies ending in spontaneous abortions before 22 weeks of gestation was not included in our database. In general, such data remain unavailable, if treated in primary health care in Finland.

We used information on purchases on psychotropic medication as a proxy of mental health disorders. Psychotropic medication is the dominating treatment approach in Finland. For instance 80% of those with an anxiety disorder using health care services for mental health reasons receive pharmacotherapy [26]. People with more severe mental health problems are more prone to seek health care [27] and thus receive prescriptions. Our proxy measure of mental ill health thus covers the majority of severe cases, but most likely will not identify all mild cases of mental ill health. A further step in the analysis would be to combine the information on purchases of psychotropic medication with hospital inpatient and outpatient data. Gathering this data both before and after pregnancy would allow analyses on the relationship between mental disorders and pregnancy in more detail.

The information on medication is more or less complete as psychotropics are not sold over the counter in Finland and practically all psychotropics are reimbursable, and thus included in our data. However, we lack information on the use of psychotropic medicine from previous prescriptions or other people's medication. We even lack information on medication given in hospital care, even though its share has diminished along the rapid deinstitutionalising of mental health services. Finally, because our data on medication were based on purchases, we do not have the exact information on whether the medication was actually ingested or not.

We were partly able to overcome the main methodological problems, which commonly complicate studies on the connection of mental health problems and induced abortion. We excluded induced abortions due to fetal reasons from our analysis. In these cases, these pregnancies are most often wanted, while most other induced abortions relate to unplanned or mistimed pregnancies. We did not have information on pregnancy intention on women whose pregnancy ended in a birth. Data from large Finnish birth cohort studies have shown that the proportion of unwanted pregnancies has decreased from 12.2% in 1966, before the current legislation on induced abortions, to 1.0% in 1985-1986 [28]. Thus, we can assume that only a small proportion of births are unwanted in contemporary Finland.

Second, we used register information on psychotropic medication prescribed by physicians. Such prescriptions are based on a medical diagnosis. We do, however, miss the women who have not seeked (mental) health care services, as well as women with mild mental disorders not requiring medication. Their proportions remain unknown in our study population, but the more severe mental disorder is, the less likely is it that the woman would have been misclassified.

Third, our register data included several important confounders, which were utilised in estimating the effect of women's age, previous pregnancy history, marital status, socio-economic status and region of residence on the risk of induced abortion. Other confounding factors can only be gathered by other means: by collecting medical records, by interviewing or by using postal questionnaires. Since this would not be feasible with such a large study material as we had, it remains unknown how much the unmeasured confounding explains our findings.

The Finnish Medication and Pregnancy database also included data on medication three months after the pregnancy. We did not use this data to evaluate mental disorders post-abortion, because of the use of tranquilising, antidepressant or psychostimulant drugs decreases significantly after birth due to labelling of these drugs as unsafe during lactation. According to the current national standards, 72 of the 180 psychotropic medicines in ATC classes N05 and N06 (40%) are labelled as possibly harmful and 50 (28%) clearly harmful during breastfeeding. Thus, all comparisons between births and induced abortions would have been biased. A longer follow-up period is needed to study the connection of pre-existing psychotropic medication and the long-term effects of pregnancy and its end on mental health. Since our data showed that women with induced abortion differ substantially from women in general, the collection of even more detailed background information would be recommended. This includes for example data on pre-existing confirmed diagnosis related to mental health, education, annual income, employment status, and social assistance, which are all available in the Finnish register system [29].

The use of psychotropic medication among young women has increased in Finland since the mid-1990s. In the general Finnish population, the number of people with at least one reimbursement for tranquilising drugs (N05) increased by 8% and that of antidepressant and psychostimulant drugs (N06) by 24% between 2000 and 2005 [30]. This trend - caused by the improved availability of new types of psychotropic medication with fewer side effects, increased identification of mental disorders and changes in help-seeking patterns - was confirmed by our data consisting of women in reproductive age.

Our nationwide data provide evidence that mental problems are more common among women who subsequently have an induced abortion, even before pregnancy. Thus, any finding of an increased post-abortion rate of mental disorders has to be contrasted against the pre-existing rate, if conclusions of the impact of induced abortion on womens mental health are to be made. Most previous studies have failed to acknowledge the high pre-existing rate, leading to biased conclusions about the impact of induced abortion on mental health.

Furthermore, we show that the excess use of psychotropic drugs among women who subsequently have an induced abortion cannot be explained solely by common background factors, such as marital status or socioeconomic position. Mental health problems severe enough to lead to the use of psychotropics are an indepedent risk factor for induced abortion. The aim to reduce the number of induced abortions is a generally accepted target for reproductive health in Finland [31]. Our results suggest that it would be important to identify actions how to promote the mental and reproductive health, especially among young women, single women and women with previous induced abortions.

The current Finnish clinical practice guideline for induced abortion does state that a post-abortion check-up visit is necessary a few weeks after the procedure to detect signs of depression and to identify the rare cases of psychosis after an induced abortion [31]. Our findings indicate that pre-existing mental disorders are more common among women who have induced abortion, and it adds justification to the current recommendation. Abiding by this recommendation may improve women's health and diminish negative health consequences post-abortion.

Finland has a lower abortion rate (9/1000 women aged 15-49 years in 2008) than the other Nordic countries on average (14/1000 in 2007) or United States (16/1000 in 2005) [32]. Even if the impact of induced abortion on mental health remains undetermined, the aim of decreasing the number of induced abortions remains important. The experience from Finland suggests that this can best be done by advancing knowledge of sexual and reproductive health and improving services related to them.

The Finnish action plan on sexual and reproductive health [33] states that sexual education should reach all children and adolescents from preschool to secondary education. Local, regional and national collaboration between different professional groups and areas should create common, cross-supportive aims for sexual education and sexual health services. These services have to be located near the clients, and if possible, as a separate youth service. The selection of contraceptives should cover all existing methods. The future aim should be that all medical examinations related to starting contraception and monitoring its use should be free-of-charge. Also a free-of-charge provision for contraceptives for young people aged under 20 has been suggested. Our results indicate that women with mental disorders and mental health problems should be especially targeted to further reduce the number of induced abortions.

Furthermore, our results indicate that the potential impact of induced abortion on women's mental health cannot be judged by comparing mental health of women with a birth to women with an induced abortion without taken into account the pre-existing mental health and vulnerability of women who choose to terminate their pregnancy.

## Conclusions

Our data show that women who had used tranquilising, antidepressant or psychostimulant medication prior to their pregnancy terminate their pregnancies more often than women without such medication. Our observations should be utilised when improving care in general practice, gynaecology and mental health services. Similar, though slightly lower risk was observed for women with no previous pregnancies. This finding suggests that the pre-existing mental health status is an essential factor, which should be taken into account in trying to explain the excess of mental disorders after an induced abortion.

## Competing interests

The authors declare that they have no competing interests.

## Authors' contributions

All authors have participated in the preparation of this article and analysis, and they have accepted its final version. MG and MA prepared the analysis plan and performed them. Both MG and MA had full access to all of the data in the study and take responsibility for the integrity of the data and the accuracy of the data.

## Pre-publication history

The pre-publication history for this paper can be accessed here:

http://www.biomedcentral.com/1471-2458/10/383/prepub
